# A Simple Model for Estimating the Kinematics of Tape-like Unstable Bases from Angular Measurements near Anchor Points

**DOI:** 10.3390/s25051632

**Published:** 2025-03-06

**Authors:** Heinz Hegi, Ralf Kredel

**Affiliations:** Institute of Sport Science, University of Bern, 3012 Bern, Switzerland; ralf.kredel@unibe.ch

**Keywords:** sensor-based, kinematics, augmented feedback, dynamic exercise, unstable surface, balance training, inertial measurement unit

## Abstract

Sensorimotor training on an unstable base of support is considered to lead to improvements in balance and coordination tasks. Here, we intend to lay the groundwork for generating cost-effective real-time kinematic feedback for coordination training on devices with an unstable base of support, such as Sensopros or slacklines, by establishing a model for estimating relevant tape kinematic data from angle measurements alone. To assess the accuracy of the model in a real-world setting, we record a convenience sample of three people performing ten exercises on the Sensopro Luna and compare the model predictions to motion capture data of the tape. The measured accuracy is reported for each target measure separately, namely the roll angle and XYZ-position of the tape segment directly below the foot. After the initial assessment of the model in its general form, we also propose how to adjust the model parameters based on preliminary measurements to adapt it to a specific setting and further improve its accuracy. The results show that the proposed method is viable for recording tape kinematic data in real-world settings, and may therefore serve as a performance indicator directly or form the basis for estimating posture and other measures related to human motor control in a more intricate training feedback system.

## 1. Introduction

Physical exercise on unstable bases of support is associated with cognitive, cardiovascular, and performance benefits in coordination and balance tasks [1,2,3,4,5,6,7,8] (although the transfer of sensorimotor capabilities to tasks on stable surfaces may be limited [9,10,11]). Training on unstable bases of support can be supported by training feedback systems that provide performance indicators for coaches, augment individual, autonomous training with feedback on execution parameters; or serve as an input device for the gamification of such training scenarios [12]. Importantly, measurement systems used in such environments should not increase the complexity of using the training equipment, but rather integrate seamlessly into the training experience, while still providing relevant feedback on execution or task performance. To that end, our goal here is to lay the foundation for a cost-effective and versatile diagnostic and training feedback system capable of estimating performance-related movement characteristics. The objective is therefore to estimate performance parameters, e.g., the kinematics of the unstable support base or the dynamics of human posture, with device-mounted sensors rather than wearable sensors. While camera-based measurement systems [13,14] might offer the highest flexibility for such a task, they are associated with significant hardware costs for the image sensors and the CPU- and/or GPU-based analysis units. On top of potentially raising privacy concerns, they are further limited by their intrinsic susceptibility to artifacts introduced by varying lighting conditions, occlusions, additional persons in the field-of-view, and geometric constraints to capture the whole scene. Inertial sensors, e.g., attached to the unstable support bases and capable of directly estimating their kinematics, seem to be a more viable approach. Instead of relying on a camera-based measurement system, we therefore intend to derive these kinematics in a way that is also compatible with versatile and cost-effective device-mounted Inertial Measurement Units (IMUs). However, it is not entirely clear which postural information or performance measures [15] can be accurately derived from such kinematics alone. For more complex postural estimates, such as knee positioning or center-of-mass information, a more detailed investigation of the relationship between the support base and body kinematics could be required. Irrespective of other relevant characteristics that are not directly related to the kinematics of the support bases due to multi-joint biomechanics, these kinematics should at least correspond well with the kinematics of the feet. As such, support base kinematics can provide the necessary data for basic applications such as step counters and various other simple performance metrics in tape exercises, as well as a rudimentary control to ensure correct exercise execution.

Systems for estimating performance-related movement features on stable bases founded on accelerometers have previously been developed [16,17]. However, these systems do not work well in settings with irregular steps [18], so direct transfer to unstable support bases is impaired. Other research tackled the task of developing measurement systems for unstable bases [13,15,19,20,21], some of which also included IMUs [22,23,24]. However, deriving position data can be challenging on unstable bases, since double integration of acceleration data suffers from strong drift [25], which cannot be tared in regular intervals because there are no extended rest phases [26] between steps. We propose estimating the position of the support base by other means. To the best of our knowledge, the approach presented here has not been disseminated previously.

**Figure 1:**
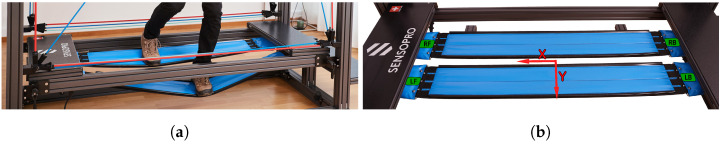
The tapes of the Sensopro Luna during a sideways exercise (**a**) and at rest (**b**), with green markings indicating the IMU positions. Blue plastic covers are screwed onto the tape, hiding the springs. The anchor points are below the black platforms at the front and back.

Specifically, our proposed setup leverages a specific constraint of slacklines and training devices with similar geometries, such as the Sensopro Luna, which consists of a metal frame and two slackline-like tapes that the exercising person is standing on (see Figure 1). Independently of whether the tape under consideration is a flexible slackline or a more rigid tape with springs on a Sensopro Luna, the tape can only take up tension (i.e., pulling forces in the direction of the tape), and so its geometry aligns with the direction of the pulling forces. The tape can thus be modeled as an idealized rope with only longitudinal geometric extension, so that one can derive the position of the contact point of a mass on the tape (which exerts another force on the rope) by only measuring the angles of the tape near the anchor points, provided the positions of the anchor points are known. So, in order to derive the position and roll angle of the tape segment directly below the mass, our model only needs the static geometric constraints of the setup and angular measurements near the tape anchor points (estimated, e.g., by IMU sensors) while deliberately ignoring potentially available measurements from segments close to the acting mass (e.g., the feet of the exercising person).

From the various potential kinematic measures of the tape, the focus in this article is on tape segment orientation and position, particularly the segment in contact with the mass. Functionally, the orientation and position axes on the tape have fundamentally different significance in tape exercises. Firstly, the longitudinal position has two potential functions: The longitudinal position of the lowest tape point approximates the foot position along the tape, which may help to ensure correct foot positioning for specific exercises. Additionally, changes in the longitudinal position of the lowest point over short periods of time might correlate to changes in pitch angle. Therefore, they also have potential for estimating the relative changes in the longitudinal position of the center of pressure (CoP) resulting from the foot pitch angle, even though the absolute pitch angle of the tape segment or foot cannot be determined in this way (a change in pitch results in a change in the longitudinal CoP position, just like moving the foot would also move the CoP position, which makes distinguishing these two effects difficult). Secondly, the lateral displacement of the support base can be utilized as a proxy for lateral CoP movement [27] and is therefore indicative of performance in balancing tasks [28]. However, depending on the geometric constraints of the training devices, it only has a very low magnitude and is consequently strongly affected by measurement noise. Even though our chosen experimental setup strongly limits its magnitude, we still include it in the analysis because of its functional relevance for lateral CoP changes and its expected larger role in slacklines due to their higher magnitude of lateral displacement. Thirdly, the vertical displacement is the most important measure for exercises with a stepping motion, since step count, intensity, and rhythm are directly derived from vertical displacement over time. Fourthly and lastly, the roll, pitch, and yaw angles of the tape segment close to the foot are related to posture and foot placement on the tape, so these angles may provide important information to correct improper exercise execution. Even if all of these angles could be measured without the model presented here by attaching an IMU sensor to the tape segment below the foot, such a setup would limit supported foot placements to a small area on the tape and increase prediction errors due to higher accelerations compared to the placement of the measurement devices close to the anchor points.

The goal of this validation study is to present a novel model for estimating tape kinematics and to assess its accuracy. After describing the proposed general model in detail and discussing a basic parameterization to further adjust it, we analyze its accuracy with both input from a motion capture system under laboratory conditions and an example IMU input that could be used under field conditions.

## 2. Materials and Methods

### 2.1. Nomenclature and Coordinate System on the Luna

To carry out our experiments, we used the Sensopro model Luna Fitness (Sensopro AG, Münsingen, Switzerland), an exercise device primarily designed for coordination training and commonly found in fitness and rehabilitation centers (see Figure 1). The Luna Fitness can be described as follows: When training on the Sensopro, users stand on two unstable tapes while receiving exercise instructions from a touch screen in front of them. Each tape consists of a rectangular non-stretchable canvas connected to two sets of four springs that are attached to the front and back anchor points on the surrounding metal frame. The total length between the front and back anchor points is 1726 mm, and the unloaded, initial length of the springs is approximately 200 mm.

We define the coordinate system as follows: The origin is in the exact center of the anchor points, between the two tapes. The *X*-axis points forwards, parallel to the long tape axis. The *Y*-axis points to the left and the *Z*-axis points upward. Roll, pitch, and yaw correspond to rotations about the *X*-, *Y*-, and *Z*-axes, respectively, using Euler angles, or, more specifically, Tait–Bryan angles following the XYZ intrinsic rotation convention. In this application, the restricted range of possible rotations of Luna tapes keeps the resulting angles similar to the corresponding axis-angle representation (which are used internally for motion capture post-processing) and helps avoid the mathematical limitations associated with Tait–Bryan angles. However, this may not be the case in other settings and should be verified before employing the same algorithm on slacklines, for example.

### 2.2. Tape Kinematics Model

**Figure 2:**
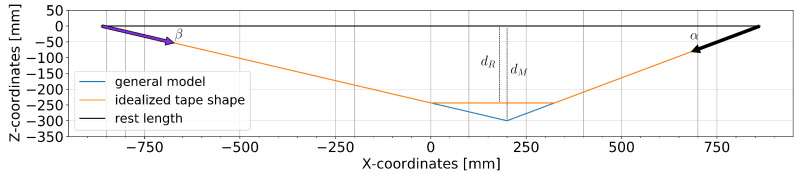
The general model for the *X*-position and *Z*-displacement $d_{M}$ compared to the actual tape displacement approximated by $d_{R}$. The black and purple arrows correspond to the front and back tape segments that determine the input angles α and β, respectively.

We consider each tape separately as an ideal rope that connects the two fixed anchor points at (±863, 0, 0) in the sagittal plane, as shown in Figure 2. The general model only requires one setting-specific parameter, namely the rest length *L*, measured from the back anchor point to the front anchor point. It treats the foot as a single point (*X*, *Z*) on the tape that causes the maximum *Z*-displacement $d_{M}$. This point is computed from the front and back angles α and β, which result in pitch-angle-based model predictions for the vertical position (*Z*) and the longitudinal position ($X_{Z}$). Note that the obtained input pitch angle values are normalized so that both the front and back angles are positive when the tape is displaced downwards; in the proper physical frame of reference, the front pitch would be negative instead. Equations (1) and (2) based on ray intersection and trigonometry, respectively, show the mathematically equivalent formulations (we noted both formulations because they can have different performance and stability properties depending on the exact implementation and system).

(1) $$\begin{array}{cccc} \; & t=\cos\left(\alpha \right)+\sin\left(\alpha \right)\frac{\cos(\beta )}{\sin(\beta )}\; & Z=-\sin\left(\alpha \right)\frac{L}{t}\; & X_{Z}=\cos\left(\alpha \right)\frac{L}{t} \end{array}$$

(2) $$\begin{array}{ccc} \; & Z=\frac{-L}{\frac{1}{\tan(\alpha )}+\frac{1}{\tan(\beta )}}\; & X_{Z}=\frac{\left|Z\right|}{\tan(\alpha )} \end{array}$$

The general model output assumes that $Z=d_{M}$ (as shown in Figure 2) and thus overestimates the actual displacement of the tape ($d_{R}$) because the foot is not a point mass. The difference between $d_{M}$ and $d_{R}$ depends on the size of the shoe, the position of the foot on the tape, the flexibility of the shoe and the foot, and the flexibility of the tape around the shoe. For example, assuming a flat segment of a length of $L_{F}=260$ mm results in a 0.85-ratio for $d_{R}$/$d_{M}$ (applying the intercept theorem results in $d_{R}/d_{M}=L_{F}/L$), which yields an alternative, parameterized model output $Z_{R85}$. Note that the longitudinal position (*X*) is not affected by this parameterization, but the correct *X*-position of $d_{R}$ could be anywhere below the shoe.

We use the same basic model in the horizontal plane to infer the yaw-angle-based model predictions for the lateral displacement (*Y*) and the longitudinal foot position ($X_{Y}$). For the yaw-angle-based model, however, we do not include an alternative parameterization correcting the foot segment length because only small *Y*-displacements are possible on the Sensopro Luna, and so the absolute error remains small, too. An additional property that the model does not consider is the fact that the springs of the Sensopro Luna can have different resistance to lateral displacement from the rest of the tape, resulting in different yaw angles along the longitudinal tape axis. Similarly, the roll rotation angle can also be modeled as increasing linearly along the *X*-axis, but suffers from the same issue because the spring segment has different resistance to angular deformation than the tapes. Contrary to the yaw-based lateral displacement, this effect is too large to ignore for the roll angles. Therefore, the following four modeling variants are included in the analysis for comparison:

- A non-configurable model expecting linearly increasing roll ($R_{M}$);
- A model with spring-coefficient parameterization expecting the rotation in the spring segment to be a fixed multiple of the rotation in the tape ($R_{S}$);
- A model with the same spring-coefficient parameterization as in $R_{S}$ but with an additional weighted sum based on the longitudinal foot position ($X_{Z}$) to rely more on the measured input roll from the sensor that is closer to the foot ($R_{WS}$);
- A trivial approach that simply adds the measured front and back roll angles, ignoring the contribution of the remaining tape segments altogether ($R_{A}$).

Overall, the model outputs are labeled as follows: $X_{Z}$ and $X_{Y}$ denote the *X*-position predictions obtained from the *Z* and *Y* models, respectively. *Y* and *Z* describe the general (non-parameterized) model predictions for the lateral and vertical displacement, respectively. $Z_{R85}$ refers to the *Z*-prediction optimized using the 0.85-ratio parameterization obtained from the theoretical shoe-correction and the synthetic recordings, while $Z_{R82}$ refers to the 0.82-ratio parameterization obtained from the full dataset recorded in this validation study (the 0.82-ratio is only included to show how demographic-specific parameterization could further improve the output, but it is possible that it constitutes overfitting).

### 2.3. Validation Study

We conducted an exploratory assessment with a convenience sample of three participants, denoted as *A*, *B*, and *C*, with shoe sizes between 42.5 and 45 (EUR shoe sizes, with shoe lengths measured at 28 cm to 32 cm and shoe widths at 11 cm to 12 cm). Each participant performed the following ten exercises on the Sensopro Luna: (01) stepping in place; (02) strong steps; (03) sprinting; (04) symmetrical bouncing; (05) one-leg stand; (06) walking back and forth; (07) walk with roll variation; (08) walk with yaw variation; (09) stepping with variation in lateral foot positioning (*Y*); (10) standing sideways (facing to the right) and stepping (Figure 1a shows the left-facing version of this exercise). Exercises 01–05 and 10 were similar to standard Sensopro exercises, but focused on variation in movements instead of consistency. Exercises 06–09 were not typical exercises, but ensured coverage of a bigger range of possible states. The exercises were performed for 60 s each, but the recordings were longer to allow for start-up and shutdown sequences. Directly after the start-up sequence and before the start of the exercise, participants jumped onto the tapes to facilitate temporal alignment of the IMU and motion capture data streams during post-processing.

**Figure 3:**
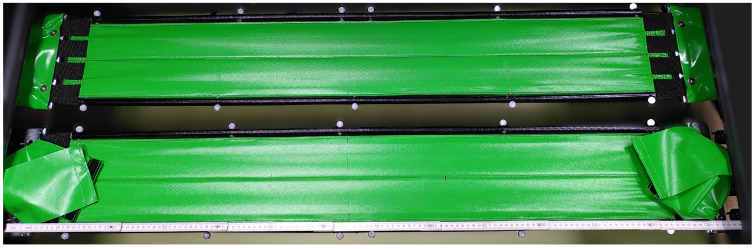
Reflective marker on the tapes (with the front on the right hand side). The plastic covers on the right tape have been loosened to partially expose the metal springs.

A Vicon optical motion capture system (10 Vicon T20s cameras, 2 MP, 500 Hz, Vicon Nexus 2.13, Vicon Motion Systems Ltd., Oxford, UK) tracked foot and tape movement by means of reflective markers (14 mm diameter). We attached three markers to each foot and eleven markers to the inner (five markers) and outer (six markers) edges of each tape, as shown in Figure 3.

The markers were grouped into eight pairs per tape, with the inner edge marker in the middle of each tape belonging to two pairs. These pairs split the tape into seven sections, with each marker pair being the border between two sections. This way, each tape section had three (middle section) or four (other sections) markers defining the section position and rotation, with the exception of the front and back sections that connected the two tape markers with the anchor points (the view of the anchor points was obstructed by the metal frame of the Luna, so the anchor point positions had to be reconstructed from static measurements and markers on the metal frame tracking potential shifts). The Tait–Bryan angles (up to this point in the calculation, angle-axis and quaternion representations were used internally for the transformations) of the front and back sections later served as inputs for the different kinematic model functions. Additionally, one IMU (SFM2, Sensor Maestros LLC, Denver, CO, USA) was attached to the bottom side of the front and back sections of each tape (four IMUs in total). Using sensor fusion of accelerometer, gyroscope, and magnetometer data, these sensors estimated the front and back section roll, pitch, and yaw angles. This setup serves as an example of a cost-effective orientation measurement system.

A third-order 100 Hz low-pass Butterworth filter was applied to the data before resampling and interpolating it from 500 Hz (resp. about 400 Hz for IMU data) down to 200 Hz, so that the resulting motion capture and IMU data shared the same timestamps. The coordinate system was then transformed to ensure that the longitudinal tape axis was exactly aligned with the *X*-axis. As a next step, the relative translations and rotations of each tape section were computed using the same transformation (these transformations were found by applying the align_vector method in the scipy.spatial.transform.Rotation class [29], which internally applies the Kabsch algorithm [30]). To improve numerical stability, our implementation of the model defaulted to zero predictions (i.e., no displacement, *X* in the center) when given small input angles (less than 0.1°). Data points where the center of the foot segment was at least 70mm above the center of the tape segment were excluded from the subsequent analyses, because this indicates that the foot was almost or entirely removed from the tape (this threshold was determined from the height of foot markers when standing still; it is still possible for the foot to be in partial contact with the tape above that threshold, for example, by tiptoeing). Similarly, data points where both feet were on the same tape, which only happened in trial 10, were also excluded. A total of 30 trials were processed for both tapes, resulting in 60 single-tape recordings. One trial (B08) was cut to only approximately 56 s instead of the regular 60 s due to a technical issue causing a delay in the IMU start-up sequence. All other trials were cut to exactly 60 s of exercise time.

### 2.4. Statistical Evaluation

For the statistical analysis, we first checked the applicability of the model and obtained a rough estimate for the application-specific model parameterization by analyzing data from separate trials where the tape had been manipulated in a more synthetic manner by hand (*Z*-displacements without roll, *Y*-displacements without *Z*-displacements, and roll without *Z*- or *Y*-displacements). This yielded the required parameters for $R_{S}$, $R_{W}S$, and $Z_{R85}$. Then, we assessed the accuracy of each model output, first for motion capture-based input angles and then for IMU-based input angles, by comparing the model predictions to the reference values, defined as follows: For each point in time, the reference *Y*-, and *Z*-displacements were taken from the tape segment with the highest absolute *Z*-displacement. The reference *X*-position was obtained from the motion capture markers attached to the foot and the reference roll angle was taken from the tape section with the highest absolute roll (this might be different from the section with the highest *Z*-displacement because the foot can be in contact with several tape sections).

For the motion capture-based input angles, we visualized the prediction error with a modified box plot, compiled a table showing the Root-Mean-Squared Error (RMSE) for each trial, and generated more detailed plots showing the effect that *X* and *Z* position have on the prediction error of key outputs ($X_{Z}$, *Y*, $Z_{R85}$, and $R_{WS}$). For the IMU-based input angles, we first plotted the difference between IMU- and motion capture-based angles for a single trial. Because of the systematic bias in pitch drift, we then applied a simple drift adjustment for pitch by shifting negative values up before visualizing the overall IMU-based prediction errors with a modified box plot again.

## 3. Results

### 3.1. Applicability of the Model

Because post-processing was kept to a minimum for the synthetic checks, the measured marker coordinates in the figures were not corrected for slight axis-misalignment, marker placing inaccuracies, or marker jitter, which might sometimes result in a few millimeters of difference between the recorded frame marker positions and anchor point coordinates.

**Figure 4:**
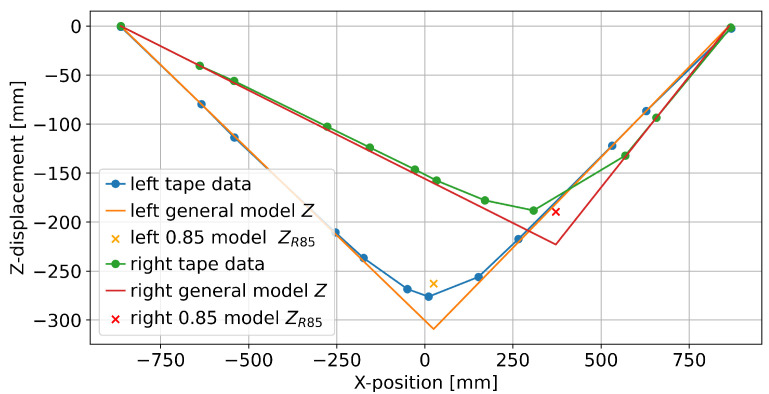
One data point of the left and right tapes in the sagittal plane (as seen from the side).

**Figure 5:**
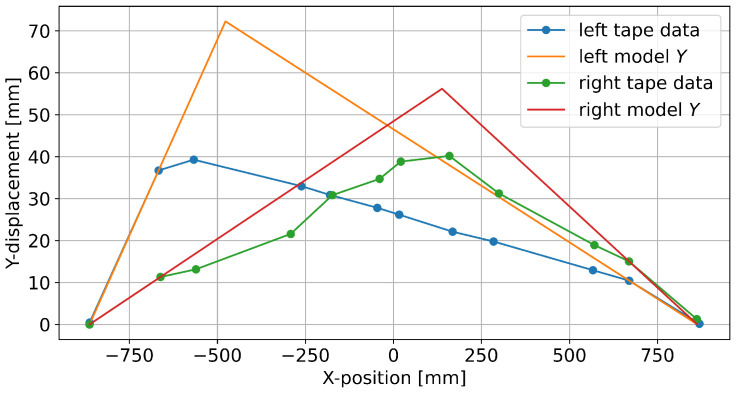
One data point of the left and right tapes in the transversal plane (as seen from above).

**Figure 6:**
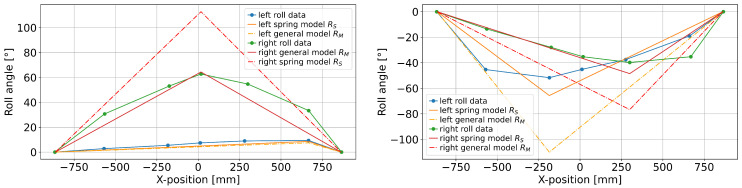
Variation in roll angle along tape axis compared to non-parameterized ($R_{M}$) and parameterized ($R_{S}$) model outputs.

Figure 4 shows a consistent pitch for tape segments that are not near the foot position, as would be expected based on the simple rope model. The model output overshoots the actual *Z*-position, which is ameliorated when employing the 0.85 shoe length factor. A similar plot is shown for lateral displacement (*Y*) in Figure 5. Note that the maximal lateral displacement is much smaller than the vertical displacement shown in Figure 4, and some markers do not lie on a straight line. The yaw angles are larger in the first and last segment compared to the more central segments. Furthermore, the model prediction near the back (i.e., the left tape in Figure 5) shows a substantially larger overshoot than the model prediction towards the center (right tape), despite similar absolute *Y*-displacements in the measured data. Finally, Figure 6 shows how the tape roll develops along the longitudinal axis (*X*), with the non-parameterized model strongly overestimating the maximum roll angle.

### 3.2. Model Accuracy

#### 3.2.1. Descriptive Statistics

A total of 359,211 data points were recorded, covering 1796 seconds of exercise time. Due to the foot being removed from the tape, 54,781 and 52,916 of these samples were excluded from the following analysis for the left and right tapes, respectively. Consequently, the analysis included 304,430 and 306,295 samples for the left and right tapes.

**Figure 7:**
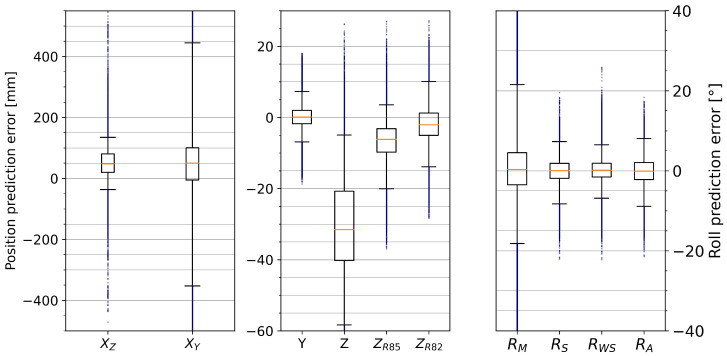
Modified box plots of the prediction errors in all samples. The whiskers range from the 2nd to the 98th percentile, and the boxes cover the 25th to 75th percentile. The median is shown as an orange line, and all outliers (highest and lowest two percentiles) are marked in blue.

Figure 7 shows the accuracy of the different model outputs. Most (±2σ, i.e., 96%) pitch-based predictions ($X_{Z}$) lie within [−4cm, +14cm] of the measured *X*-position of the foot, but the yaw-based predictions ($X_{Y}$) are spread out to [−36cm, +45cm]. Both *X*-predictions also have notable outliers exceeding ±40cm (an interval that covers more than half the tape length). Most predictions for lateral displacement (*Y*) are within an error margin of ±8mm (note, however, that the maximum observed lateral displacements in these trials were only ±70mm). For the vertical displacements, the parameterized solutions resulted in more accurate predictions than the non-parameterized model (*Z*). The median of the prediction error for the 0.82-ratio is closer to zero compared to the 0.85-ratio, but the variation remains similar: for $Z_{R85}$, more than 96% of predicted positions are within −8±12mm of the actual displacement, while $Z_{R82}$ moves that interval to 2±12mm. Finally, the spring-corrected model predictions ($R_{S}$) lie within ±8.5°, except for outliers, which is also more accurate than the non-parameterized roll angle model output ($R_{M}$). Adding weights based on the reference *X*-position has a small positive effect, with the weighted spring-corrected roll predictions ($R_{WS}$) lying within ±7° of the reference for over 96% of all data points. The simple approach $R_{A}$ is only slightly less accurate, with ±9°. Similarly, half of all predictions lie within ±1.9° of the reference for $R_{S}$ and $R_{WS}$, while the same proportion covers the interval [−2.2°, +2.0°] for $R_{A}$.

#### 3.2.2. Effect of Position on Accuracy

**Figure 8:**
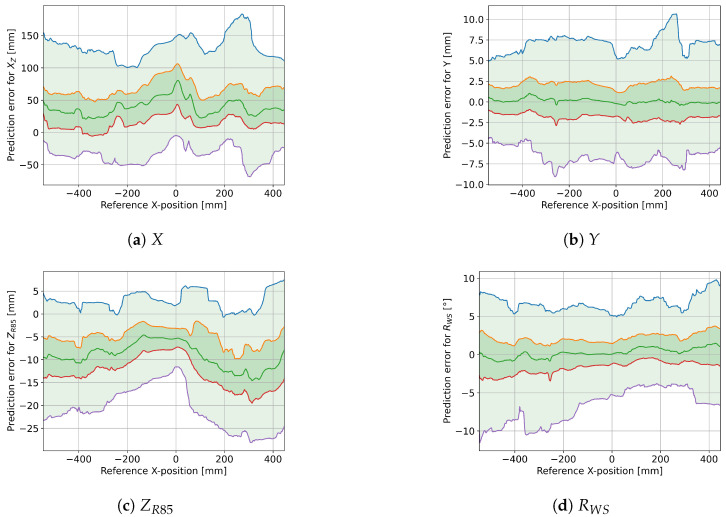
Effect of X-position on prediction error for *X*, *Y*, $Z_{R85}$, and $R_{WS}$. The green line is the median, the dark green area covers the 25th to 75th percentiles, and the light green area covers the 2nd to 98th percentiles (96% of all data points). The blue, orange, red, and purple lines correspond to the 98th, 75th, 25th, and 2nd percentiles, respectively.

**Figure 9:**
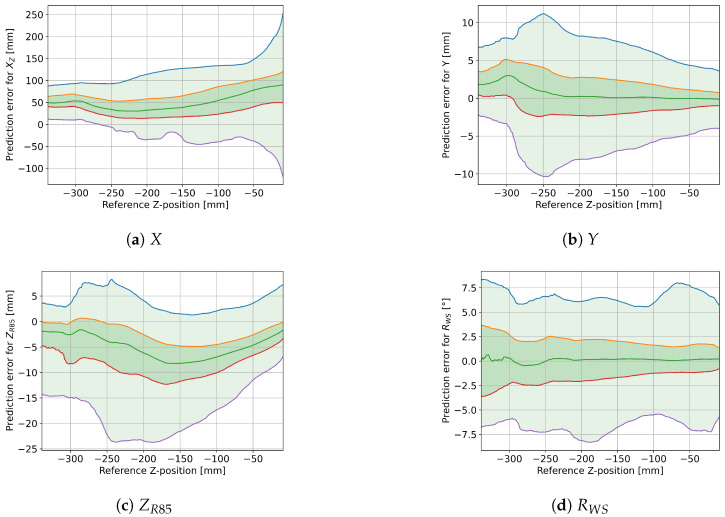
Effect of Z-position on prediction error for *X*, *Y*, $Z_{R85}$, and $R_{WS}$. The green line is the median, the dark green area covers the 25th to 75th percentiles, and the light green area covers the 2nd to 98th percentiles (96% of all data points). The blue, orange, red, and purple lines correspond to the 98th, 75th, 25th, and 2nd percentiles, respectively.

Figure 8 shows how the different model outputs are affected by the foot position along the tape axis. The spring-corrected roll angle displays a tendency to have smaller prediction errors towards the center of the tape. A similar pattern emerges in $Z_{R85}$, but less pronounced. The predictions for longitudinal (*X*) and lateral (*Y*) displacements both show variations with no clear pattern. Figure 9 similarly shows the effect of the downward displacement of the tape. Other than at small (≤5 cm) and at very big (≥30 cm) displacements, there seems to be little effect on the prediction error, except some curvature in $Z_{R85}$.

Table 1 shows how the accuracy is affected by different exercises. Notably, the RMSE of the longitudinal position prediction $X_{Z}$ is lower in sideways trials and highest in sprinting trials. The lateral (*Y*) and vertical (*Z*) displacement outputs show largely consistent performance over the different trials, except for increased RMSE for $Z_{R85}$ and $Z_{R82}$ in the walking trials with increased *X*-position variation (trials 06–08). The roll angle predictions also seem to have higher RMSE in these walking trials, but they also have higher RMSE in trials 04 and 05 (bouncing and one-leg stand), with trial 10 (sideways) only having an above average RMSE for $R_{M}$ and $R_{WS}$.

### 3.3. Model Accuracy on IMU Data

**Figure 10:**
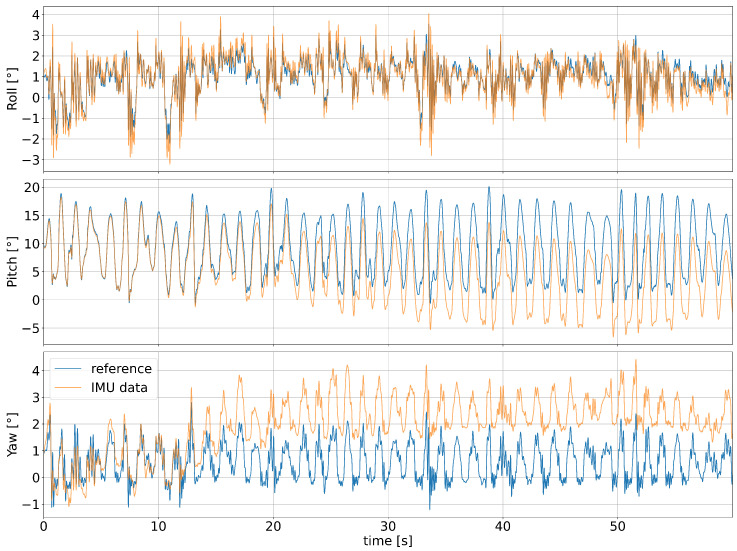
Comparison between IMU angles and reference motion capture data for a single trial (C02).

**Figure 11:**
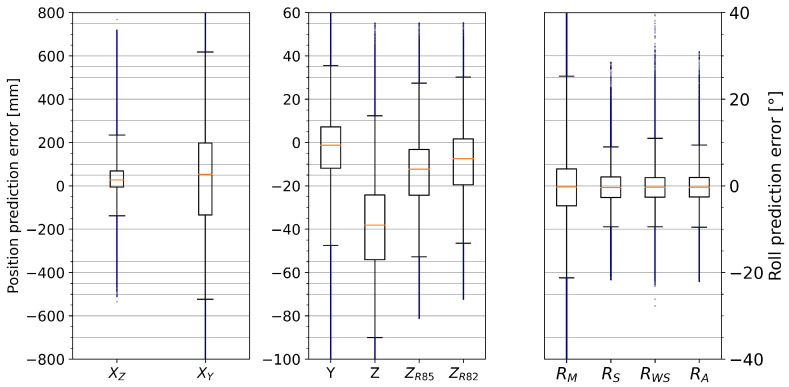
Modified box plots of prediction errors using IMU input data. The whiskers range from the 2nd to the 98th percentile.

Figure 10 shows that the IMU pitch and yaw angles used as inputs for the model predictions drift away from the reference angles recorded by the motion capture system, but this is not the case for the roll angles. Figure 11 shows that the IMU-based predictions for *X*, *Y*, and *Z* are much less accurate than the ones obtained from the motion capture system. The roll predictions are similar to the one based on motion capture data. Note that the prediction error after the drift for pitch angles was corrected with the assumption that the tape cannot exceed its rest height, i.e., that the input angle for *Z* and $X_{Z}$ is always positive. Compared to the motion capture based model output, the 96% prediction error interval increased as follows: from [−4 cm, +14 cm] to [−14 cm, +24 cm] for $X_{Z}$; from ±8mm to [−5 cm, +4 cm] for *Y*; from −8±12mm to [−53 cm, 27 cm] for $Z_{R85}$; and from ±7° to [−10°, +11°] for $R_{S}$, $R_{WS}$, and $R_{A}$. Notably, $R_{WS}$ had a larger prediction error than $R_{A}$ and $R_{S}$, which was not the case for the predictions based on motion capture data.

## 4. Discussion

### 4.1. Summary of Findings

The proposed model for estimating relevant kinematic data for exercises on tapes was successfully applied to the Sensopro Luna Fitness, including a simple parameterization for foot-size adjustments. With near-perfect input angles from the motion capture system, the model achieved a prediction error within a few centimeters of the reference measurements for lateral (*Y*) and vertical (*Z*) displacements (see Figure 7). Since the tape is wide enough to wrap around the foot and the reference system could only measure marker positions near the edges of the tape, this may at least partially lie within the measurement error for the reference data. Similarly, the error range for the longitudinal position (*X*, excluding 4% of data points as possible outliers) is smaller than the length of the foot. While this seems like a large variation at first, the following aspect needs to be taken into account before evaluation: by shifting the center of pressure forward and backward, the theoretically perfect *X* measurement (i.e., the CoP position) would also shift while barely affecting the reference *X*-position that is only based on the foot markers. This interpretation is supported by the fact that the sideways trial (exercise 10) had a noticeably smaller RMSE than the other trials (see Table 1). The same model does not perform as well for roll angle predictions; even the parameterized and weighted versions are not substantially more accurate (see Figure 7) than a simple addition of roll angles measured at the front and back. Finally, while the estimation based on IMU measurements showed higher prediction errors than the motion capture-based predictions, the accuracy would probably suffice for some applications, such as gamification. However, there is still room for further improvements in several areas.

### 4.2. IMU Drift

Drift in the IMU angles could be mitigated in several ways. One simple adjustment was already included here, namely prohibiting negative pitch input angles. This brought the $Z_{R85}$ prediction error down from [−35mm, 61mm] to the [−53mm, 27mm] shown in Figure 11, and reduced $X_{Z}$ from [−33cm, 42cm] to [−14cm, 24cm]. Another strategy would be to detrend the signal to mitigate the effects of drift (from short experiments, a third-order 0.001 Hz high-pass Butterworth filter seems to work well for pitch angles; similarly, a 0.01 Hz high-pass filter seems adequate for roll angles), but this has the downside of hiding long-term shifts in movement patterns over the exercise duration. When additional information about the intended exercise is provided (e.g., by linking the data collection and algorithms to the selected exercise or by implementing an automatic classification system similar to [24]), this could be used to impose more specific constraints on the input data. For example, in symmetrical exercises where the whole weight is on the tapes, the displacement averaged over a time window of several seconds should remain more or less constant. Furthermore, some part of the pitch angle drift seems to be caused by the repeated up and down movements during regular exercise or the tape swinging freely in its natural frequency. The one-sidedness of the observed pitch drift patterns could therefore be explained by the general sensor fusion algorithm considering accelerations near 1G as resting points for the internal drift correction. This would lead to the inclusion of time frames with 1G downward accelerations (in addition to the 1G upward accelerations expected at rest). Overall, it would likely be beneficial to implement a specialized sensor fusion algorithm that would estimate the input angles based on the raw accelerometer, gyroscope, and magnetometer data. By adapting the sensor fusion algorithm to the specific setting, in which the state space of possible angles is severely restricted, such drift patterns could potentially be detected and avoided. For example, when the foot contact is suddenly removed, Sensopro Luna tapes show fairly regular oscillation frequencies that could be filtered in the measured input angles to avoid drift and increase reliability.

### 4.3. Outlier Detection

In addition to these possible improvements to the IMU input, the outliers could be detected independently of the orientation measurement system. Our model currently only considers each time frame separately. Generally, temporal coherence conditions can be enforced on *X*, *Y*, and *Z* to reject at least some of the observed outliers, because these values should change smoothly. Also, since the model outputs two variables for each data point ($\left(X_{Z},Z\right)$ or $\left(X_{Y},Y\right)$), we can infer information about the reliability of one by using the other. Low *Z* values generally lead to unreliable $X_{Z}$ estimations, and the same is true for *Y* and $X_{Y}$. Conversely, moving the foot in the *X* direction usually involves lifting the foot off the tape. So, large changes in *X*-predictions (i.e., larger than the foot length) without an intermediate foot-lift-off phase are likely inaccurate (unless for exercises where both feet are on the same tape).

The biggest outliers for the *X*-position tend to happen when the tape is not under load, because then, even little changes in input angles can have big effects on the predicted $X_{Z}$. When removing the foot from the tape, it quickly oscillates up and down in a range of about ±4 cm. It would be best to detect idle or oscillating tape conditions and handle them separately to avoid these issues. For a similar reason, the yaw-angle-based longitudinal position estimation ($X_{Y}$) does not seem to be a viable option for the Sensopro Luna; displacements in the *Y* direction are in the range of a few centimeters, and small perturbations in the input will therefore have a big effect on the predicted $X_{Y}$-position. For this reason, slight marker shifts and tape deformations during recording are also sufficient for explaining the chaotic lines observed in Figure 5, since the marker positions only deviate a few millimeters from a straight line (excluding the first and last segments). When using IMUs for tape angle measurements, the predicted *Y* and $X_{Y}$ positions would be even less accurate because the yaw angle required as n input is more susceptible to drift and fluctuations (contrary to pitch and roll, the sensor fusion for yaw angles cannot use gravitational acceleration for drift correction and must rely on magnetometer data instead). These issues can generally be mitigated by rejecting measurements with small angles altogether. This is a reasonable procedure under real-world conditions since the *X*-position is a meaningless measure when the foot is removed from the tape and since we are not interested in the vertical and lateral tape positions when unloaded.

### 4.4. Effects of X- and Z-Position on Accuracy

The effects of the *X*- and *Z*-positions on the different model outputs shown in Figure 8 and Figure 9 have a few different possible explanations:

- Generally, more data samples are gathered near the center of the tape (i.e., *X* near 0), because the standard exercises in the first five trials have little *X*-variation. It is possible that the movement in the latter five trials with more *X*-variation is also more erratic, which would generally increase the prediction error. Furthermore, the motion capture markers are closer together near the center of the tape, so the reference measurements might be less accurate in the front and back sections for all variables other than the *X*-positions.
- The longitudinal position prediction $X_{Z}$ defaults back to the center of the tape when the tape is not under load, which would explain the larger variations for small *Z*-values and possible smaller variations when the *X*-reference is near zero. Since the variation is mostly within a [−10cm,+20cm] interval around the reference *X*-position, it is possible that this variation is not due to prediction errors at all: the distances of heel-markers and toe-markers to the foot-center position are also about 10cm and 20cm, respectively, so this could be due to changes in the center-of-pressure position (which is what the model actually tries to predict) relative to the foot-center position.
- Larger *Y*-variation can be achieved by the user when positioned near the center of the tape (*X* near zero) and with increased downward force applied to the tape, at least up to a point, since extreme *Z*-displacements are difficult to achieve when there is additional sideways displacement. With larger real *Y* variation, larger prediction errors are to be expected.
- There is less variation in $Z_{R85}$ prediction error near the center of the tape (see Figure 8c), but the pattern is not symmetrical, so this could be an instance where the more erratic movements in the trials with more *X*-variation (i.e., trials 06–10) affect the prediction error. The curved form with increased *Z*-displacement up to −250mm could be indicative of a non-linear error term that has not been included in the parameterized model, especially since the median error is affected the same way.
- The distribution of the roll angle prediction error seems to be more spread out for smaller *Z*-displacements, but the 25th to 75th percentile ranges show the exact opposite effect. This can, again, be explained by smaller variations in roll angles for extreme *Z*-values. However, there is a much stronger dependency on the *X*-position: increased distance from the center leads to up to three times larger prediction errors. Consequently, the current implementation of the parameterized model is not suitable for *X*-positions near the very front and back of the tape.
- According to Figure 8, the prediction error is not symmetrical in the sagittal axis. This could be explained by the fact that in most trials, the feet were pointing forward, so that the toes would have a higher X-value than the heels. The only exception was trial number 10, where the feet were turned to the right, resulting in a smaller effective foot segment. Generally, the prediction error for $Z_{R85}$ and $R_{WS}$ seems to increase with larger distance from the center, but it is not clear whether this is due to a potential limitation of the model, due to the feet affecting the measured angles when closer to the anchor points, or just due to less regular movement patterns in the trials that also had large movements in the X-direction. However, if the different movement patterns are the actual reason for the increased prediction errors, we would expect a noticeable pattern in the trial RMSE in Table 1.

### 4.5. Limitations

The accuracy assessment conducted in this study is limited by the measurement setup in several ways, some of which have already been mentioned before. Here, we list the most pertinent caveats: First, the validation study is only meant as an assessment of the model accuracy, with a focus on covering basic exercises as well as a large variety of tape poses. It is by no means a representative study for making statements about general exercise patterns and human body kinematics during training. Furthermore, it is possible that the reported accuracy ranges exhibit specific biases, so additional setting- and exercise-specific validation with more participants is recommended before the model is used in practice. Second, only small *Y*-variations were achieved, potentially reducing the observed prediction error independently of the chosen model. Third, we used foot markers to detect and exclude measurements where the foot was not on the tape. Under real-world conditions, this would have to be detected algorithmically if these data points needed to be excluded. Finally, the theoretically ideal reference position would be the center of pressure, which cannot be determined directly in our setup—its position may be anywhere below the foot (with a larger range of lateral variation than in slacklines [27]). Moreover, marker positions and complex tape behavior further separate the measured values from the ideal CoP position. Nevertheless, these inaccuracies should lie in an inconsequential range for everything but the *Y*-variations, and so we believe that this validation setup is sufficient to show the usefulness of the model. Removing these unsystematic measurement errors in the data (if possible at all) could affect the estimated accuracy in both directions, so it is not clear whether it would increase or decrease the estimated accuracy ranges.

One general limitation of the model presented here is that we expect the tape to be loaded, which is not always the case. When the mass is quickly removed from the tape, this can lead to complex oscillations that affect the orientation at the front and back. These oscillations can even lead to the inputs having different signs, which leads to numerical instability for all model outputs. Even if both angles have the same sign, it is possible that the two 3D rays resulting from tape angle measurements do not intersect. Our proposed method looks at the XZ and the XY 2D-planes separately to determine the $\left(X_{Z},Z\right)$ and $\left(X_{Y},Y\right)$ intersection positions, respectively. If there is no 3D ray intersection, then this will result in $X_{Z}\neq X_{Y}$, which could serve as an indicator for inaccurate measurements. However, the *Y* displacements we observed here were not large enough to make use of this. Generally, if IMU measurements result in a discrepancy between $X_{Z}$ and $X_{Y}$, we suggest giving more weight to the $\left(X_{Z},Z\right)$ position due to the yaw angle being less reliable.

The model also does not account for the change in spring length under load, which affects the exact *X*-position at which the roll angle is measured. Therefore, the exact factor relating measured roll to foot segment roll may change with increased pitch (although we would expect a more clearly visible pattern in Figure 9d if this effect was strong).

### 4.6. Future Research

Future research should try to replicate these accuracy assessments for slacklines. The observable parameter ranges are severely restricted by the Sensopro Luna as follows: *Y* is within ±7cm, *Z* is between 0cm and 38cm, and roll is between −30° and 30°. An assessment in slacklines would therefore be especially important for the *Y*-displacement, but potentially benefit the other parameters as well since it would allow for larger displacements in all directions (e.g., [20] used a slackline of 3 m length and 5 cm width, compared to the 1.73 m long and 20 cm wide tapes used here). Additionally, a slackline would have one homogeneous material throughout the full length of the elastic ribbon, so it may be beneficial to investigate the potentially simpler relationship of these quantities with measurements in slacklines, too. For example, given a yaw and roll angle estimation formula on slacklines that is parameterized with length and rotational stiffness constants, another candidate for computations on Sensopro tapes could be found by applying that same formula twice (with different parameter values on the spring and tape segments). Alternatively, setting-specific heuristic approaches like the one applied in $R_{A}$ could already be sufficient, but those likely also benefit from data-driven adjustments.

Another interesting topic for further investigation is the relationship between model output and kinetic features, specifically between foot pitch, center of pressure, and predicted *X*-position. We expect that some variation in the $X_{Z}$ prediction can be explained by changes in posture affecting the exact center-of-pressure position beneath the foot and, consequently, the position of the lowest point of the tape, rather than solely being due to measurement or model errors. If that is the case, it would be possible that the pitch or the center-of-pressure *X*-position could be predicted if the exact foot placement on the tape is given. The feet are generally not completely removed during typical exercises, so the *X*-position of each foot should be fixed in some sense, which would make these relative variations useful in practice. While we already saw some correlation between small *X*-variations and foot pitch variation in this dataset, a different measurement setup would be required to identify a direct relationship between these quantities with certainty. For example, leveraging in-sole pressure measurement devices or employing a full-body motion capture system with biomechanical modeling, one could potentially relate variations in model predictions to center-of-mass and center-of-pressure movements.

Finally, although we focused on the tapes of the Sensopro Luna in the accuracy assessments and proposed further adjustments based on slackline measurements, we see the simplicity of the general model as a potential strength in the sense that it could already be applied to other Sensopro models and slacklines as-is, even without a more detailed investigation into the best possible setting-specific parameterization. Nevertheless, we recommend a previous application-oriented validation in order to obtain pertinent accuracy ranges and updated parameter values, since the accuracy reported here may otherwise not apply due to population biases and different exercise sets. It would also be interesting to see how such a model would perform for trampolines, where several input angles could be measured at different positions. It may be possible to adapt this approach to trampolines by either treating them as several overlapping two-dimensional rope models or by expanding the approach to a full three-dimensional model of unstable bases of support.

## 5. Conclusions

The general model provides a rough estimate of the most relevant kinematic parameters, sufficient for gamification applications. Adjusting the model with a few tape-specific parameters greatly reduces bias and improves the accuracy of the model. With these adjustments, generating feedback for coordination training based on the output of this model seems possible. Our results show moderate accuracy for sagittal foot positioning along the tape and high accuracy for vertical displacement, while lateral displacements, roll angles, and potential kinetic relationships may require further investigation. IMU-based measurements suffer from drift over time, but appropriate drift corrections can mitigate this issue. Promising related applications of the proposed model include slacklines and trampolines.

## Figures and Tables

**Table 1:** RMSE of prediction error for each exercise.

Exercise	$X_{Z}$	$X_{Y}$	*Y*	*Z*	$Z_{R85}$	$Z_{R82}$	$R_{M}$	$R_{S}$	$R_{\mathrm{WS}}$	$R_{A}$
	(mm)	(mm)	(mm)	(mm)	(mm)	(mm)	(°)	(°)	(°)	(°)
01	80.7	94.7	2.5	30.1	5.0	4.6	4.8	2.2	1.8	2.7
02	79.0	108.1	2.2	33.3	5.5	5.2	4.3	1.8	2.1	2.0
03	90.3	115.0	2.7	32.7	7.1	3.7	4.4	1.7	2.1	1.9
04	58.8	103.5	3.7	33.7	9.0	4.7	6.6	5.0	3.3	5.5
05	60.0	112.6	3.3	36.8	8.5	5.1	7.0	4.8	3.5	5.5
06	65.5	205.8	2.9	38.5	12.0	7.8	8.4	2.5	2.6	2.5
07	64.8	203.1	3.8	33.8	11.5	7.8	12.2	3.7	3.6	3.9
08	56.8	196.7	3.4	35.2	11.4	7.6	13.4	4.3	4.0	4.6
09	59.9	117.5	4.3	33.0	6.7	4.4	8.3	4.0	3.4	4.7
10	23.6	179.4	3.0	30.3	7.0	5.7	10.9	2.5	3.2	2.4
all	66.6	148.8	3.3	33.7	8.7	5.8	8.6	3.5	3.0	3.8

## Data Availability

The raw data supporting the conclusions of this article will be made available by the authors on request.

## Abbreviations

The following abbreviations are used in this manuscript:

IMU	Inertial Measurement Unit
CoP	Center of pressure
RMSE	Root-Mean-Squared Error
